# The effect of long-term cigarette smoking on selected skin barrier proteins and lipids

**DOI:** 10.1038/s41598-023-38178-7

**Published:** 2023-07-18

**Authors:** Kristýna Hergesell, Anna Paraskevopoulou, Lukáš Opálka, Vladimír Velebný, Kateřina Vávrová, Iva Dolečková

**Affiliations:** 1 Skin Barrier Research Group, Faculty of Pharmacy, Charles University, Hradec Králové, Czech Republic; 2 Contipro a.s., Dolní Dobrouč 401, 561 02 Dolní Dobrouč, Czech Republic

**Keywords:** Biochemistry, Biological techniques, Cell biology, Physiology, Pathogenesis

## Abstract

The negative impact of cigarette smoking on the skin includes accelerated aging, pigmentation disorders, and impaired wound healing, but its effect on the skin barrier is not completely understood. Here, we studied the changes in selected epidermal proteins and lipids between smokers (45–66 years, smoking > 10 years, > 10 cigarettes per day) and non-smokers. Volar forearm epidermal and stratum corneum samples, obtained by suction blister and tape stripping, respectively, showed increased thickness in smokers. In the epidermis of smokers, we observed a significant upregulation of filaggrin, loricrin, and a trend of increased involucrin but no differences were found in the case of transglutaminase 1 and kallikrein-related peptidase 7, on the gene and protein levels. No significant changes were observed in the major skin barrier lipids, except for increased cholesterol sulfate in smokers. Liquid chromatography coupled with mass spectrometry revealed shorter acyl chains in ceramides, and an increased proportion of sphingosine and 6-hydroxysphingosine ceramides (with C4 *trans*-double bond) over dihydrosphingosine and phytosphingosine ceramides in smokers, suggesting altered desaturase 1 activity. Smokers had more ordered lipid chains found by infrared spectroscopy. In conclusion, cigarette smoking perturbs the homeostasis of the barrier proteins and lipids even at a site not directly exposed to smoke.

## Introduction

Cigarette smoke is a complex aerosol containing condensed liquid droplets (particulate matter or tar), volatile and semivolatile compounds and gases. Besides nicotine, a highly addictive drug, it contains a number of toxic substances including polycyclic aromatic hydrocarbons (PAHs, e.g. benzo[a]pyrene), nitrosamines, aldehydes, carbon monoxide, hydrogen cyanide, etc.^1^. Cigarette smoking is one of the most popular forms of recreational drug use, strongly associated with a higher risk of pulmonary and cardiovascular diseases, diabetes and cancer. The long-term cigarette smoking also causes structural and compositional changes in the skin associated with an accelerated aging phenotype, deep wrinkles, loss of elasticity, pigmented spots, sallow coloration, and impaired wound healing^2^.

The impact of long-term cigarette smoking on the skin barrier function has also been discussed although with no clear consensus. Several studies examined transepidermal water loss (TEWL), one of the most common parameters used for the assessment of the overall skin barrier function^3^. Some of them found increased TEWL in smokers^4^, and in mice^5^ or skin explants^6^ exposed to cigarette smoke whereas others observed no significant differences in TEWL between smokers and non-smokers^7, 8^. Systematic reviews and meta-analyses showed that cigarette smoking is a risk factor for various skin diseases associated with skin barrier impairment such as atopic dermatitis^9^ or psoriasis^10^ supporting the link between cigarette smoking and skin barrier damage.

Even less is known about the mechanism of action of cigarette smoking on the skin barrier components. The skin barrier function is provided mainly by the stratum corneum (SC) composed of anucleated corneocytes surrounded by extracellular lipids. During terminal differentiation of keratinocytes, skin barrier proteins such as filaggrin, loricrin and involucrin are concentrated into the cornified envelope, a layer gradually replacing the cytoplasmic membrane of keratinocytes and forming a template for the arrangement of the extracellular lipids^11^. The cornified envelope proteins are extensively crosslinked by transglutaminases (TGs) stabilizing the cornified envelope structure^11^. Mechanical resistance of SC is provided by the corneodesmosomes tightly binding the corneocytes together. In the uppermost layers of the SC, these intercellular junctions are proteolytically degraded to allow desquamation. Among the key proteases involved in this process are kallikrein-related peptidases (KLKs) such as chymotrypsin-like KLK7 and the trypsin-like KLK5, plasmin and urokinase^12^. The corneocytes are embedded in the lipid extracellular matrix forming highly ordered, lamellar structures. Major classes of SC barrier lipids: ceramides, free fatty acids and cholesterol are present in an approximately equimolar ratio here (after a recalculation to weight, ceramides represent 50 wt%, cholesterol 25 wt% and free fatty acids 10 wt%) and they are accompanied by minor classes such as cholesterol sulfate. The barrier lipid precursors include sphingomyelins, glucosylceramides and phospholipids^11^.

The aim of this study was to elucidate the long-term effect of cigarette smoking on the skin barrier components by comparing the differences between the epidermis of smoking and non-smoking women. The samples of the epidermis and SC were obtained from volar forearms, where the influence of other factors such as UV radiation or direct exposure to secondhand smoke from burning cigarettes and exhalation is minimized. Therefore, this area should reflect primarily the systemic effect of smoking. In these samples, we evaluated the expression of the representative skin barrier proteins (filaggrin, loricrin, involucrin, TG1, KLK7), the thickness of the cellular epidermis and SC, and determined the composition and organization of the skin barrier lipids.

## Materials and methods

### Volunteers

This observational, non-interventional, cross-sectional study was approved by the ethical committee of Contipro a.s. and was in accordance with the WMA Helsinki Declaration. 16 Caucasian women from Central Europe were included in this study. All subjects gave written informed consent. Of the 16 volunteers, 8 were smokers (45–66 years, > 10 years history of smoking, > 10 cigarettes per day) and 8 were non-smokers of similar age (45–69 years, no smoking history). All the volunteers were healthy, with no evidence of any acute or chronic skin disease.

### Epidermal and SC sampling

Suction-blister technique was performed as described previously (Svoboda et al.^13^) with slight modifications. Briefly, 2 mL syringe with the inner diameter of 0.9 cm and with the front part cut was placed on the skin of volar forearm. The plunger of the syringe was pulled to induce negative pressure (approx. 250 mmHg determined previously^13^ and after the skin was sucked into the syringe (approx. 1 cm), the position of plunger was fixed using pipette tips. After blister formation (approx. 1 h) the separated epidermis was cut by scissors and stored at − 80 °C. Two samples from each volunteer were taken. The first sample was used for the lipid analyses. The second sample was divided into two halves, the first half was used for RNA isolation and the other half for immunofluorescence staining.

Tape-stripping was performed as described previously^13^ by application of 6 D-Squame sampling discs (1.4 cm, CuDerm, USA) onto the same spots of the skin. The first two discs were discarded. This process was repeated on four adjacent places of the volar forearm skin. The discs were stored at − 80 °C for further analyses.

### Quantitative real-time RT-PCR (qRT-PCR)

qRT-PCR in the samples of the epidermis was performed according to Hergesell et al.^41^. Briefly, the epidermis collected by suction blister was homogenized and the total RNA was isolated. The reverse transcription reaction was then carried out using High Capacity RNA to cDNA Kit (Invitrogen, USA) and followed by qPCR performed with specific TaqMan gene expression assays for *FLG*, *LOR*, *IVL*, *KLK7*, *TGM1*, and *RPL13A* genes (all ThermoFisher Scientific, USA) according to the manufacturer’s recommendations. *RPL13A* was used as a reference gene. The data were analyzed using the 2^−ΔΔCt^ method. For further details, see Supplementary.

### Immunofluorescence staining

The epidermis samples collected by suction blister were fixed, embedded in paraffin and cross-sectioned. After deparaffinization, antigen retrieval and permeabilization, the samples were blocked and probed with the rabbit polyclonal antibodies against loricrin, involucrin (both Abcam, UK), filaggrin, KLK7 and TG1 (all three Invitrogen, USA) and appropriate anti-rabbit secondary antibody conjugated with Alexa Fluor 555 (Invitrogen, USA). The samples were mounted in a Prolong Diamond with DAPI (ThermoFisher Scientific, USA). The images were acquired by a Leica TCS SP8 X confocal microscope (Leica Microsystems, Germany) and the fluorescence intensity quantification was performed using a FiJi software^14^. For further details, see Supplementary.

### Measurement of the thickness of the cellular epidermis and SC

The deparaffinized histological cross-sections prepared as described in immunofluorescence section above were stained by hematoxylin and eosin and the images were acquired using a light microscope. Measurement of the cellular epidermis and SC thickness was performed in 10 distinct places per sample in NIS elements program (Nikon, USA).

### Fourier transform infrared spectroscopy of lipids

Infrared spectroscopy was performed as described previously^15^ with minor modifications. Infrared spectra of the suction blister epidermal samples were measured on a Nicolet 6700 FT-IR spectrometer (ThermoFisher Scientific, USA) equipped with a single-reflection MIRacle ATR (PIKE Technologies, USA) attenuated total reflectance germanium crystal at 23 °C. All the spectra were generated by the co-addition of 128 scans recorded at a 2 cm^−1^ resolution. The analysis was performed using Bruker OPUS software (Bruker, USA). The exact peak positions were then determined from the second derivative spectra and by a peak fitting if needed. For each sample, the spectra were measured at two different areas and averaged.

### High-performance thin-layer chromatography (HPTLC) of lipids

For details, see Supplementary.

### Liquid chromatography coupled with tandem mass spectrometry (LC–MS^2^) of SC lipids

For details, see Supplementary.

### Statistical analysis

The data represent means ± SD, normal distribution of data was confirmed by Kolmogorov–Smirnov test. Unpaired T-test was used for the statistical analysis, asterisks indicate statistically significant differences between smokers and non-smokers at the following levels: *p ≤ 0.05, **p ≤ 0.01, ***p ≤ 0.001.

### Ethical approval

All procedures performed in studies involving human participants were in accordance with the ethical standards of the Contipro a.s. committee and with the 1964 Helsinki declaration and its later amendments or comparable ethical standard.

## Results

### Selected genes and proteins associated with the skin barrier function

**Figure 1:**
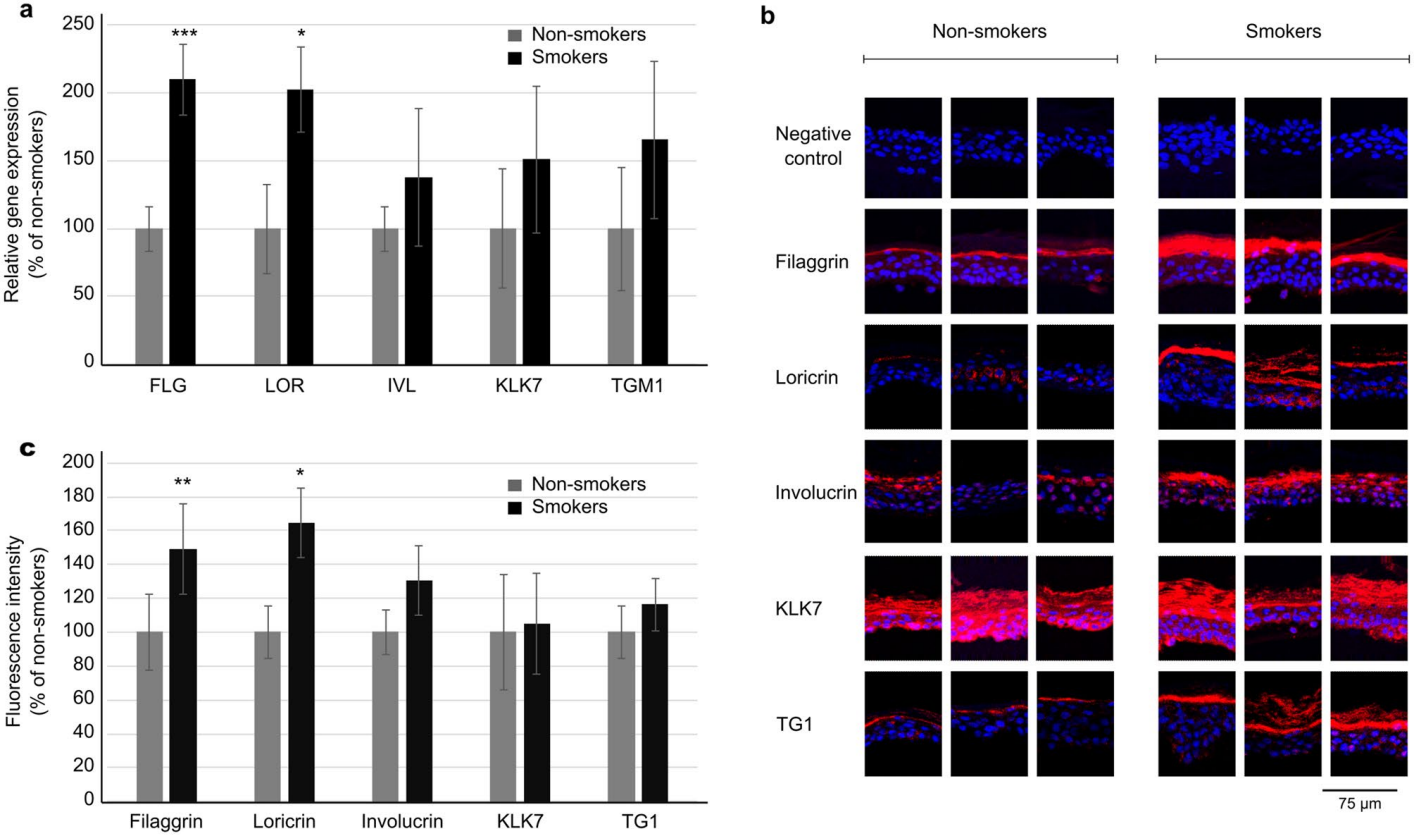
Evaluation of the selected proteins associated with the skin barrier function in the epidermis of smokers and non-smokers. (**a**) Gene expression evaluated by qRT-PCR. (**b**) Protein level evaluated by immunofluorescence staining: representative images of the stained epidermal samples from three smokers and non-smokers (red: protein of interest, blue: cell nuclei stained with DAPI), c quantification of the fluorescence intensity.

**Figure 2:**
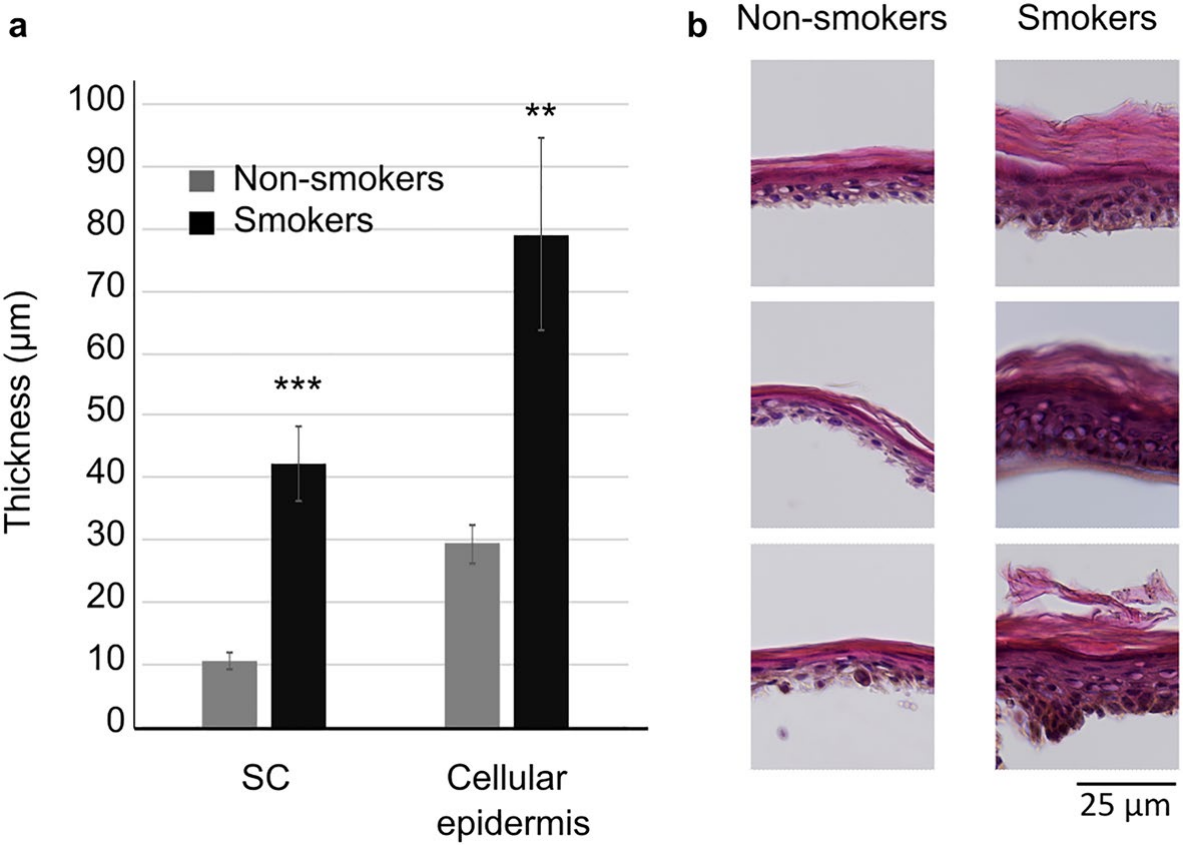
Histologic evaluation of the SC and cellular epidermis thickness in smokers and non-smokers. (**a**) Quantification, (**b**) representative images of the epidermal samples stained with hematoxylin and eosin from three smokers and non-smokers.

The results showed that filaggrin and loricrin were significantly upregulated in the skin of smokers at both the gene expression and protein levels (Fig. 1). We also observed a trend of increased gene expression of involucrin and TG1 which correlated with their elevated protein levels in the epidermis of smokers, although these results did not reach statistical significance. A trend toward higher gene expression of KLK7 was also found in the epidermis of smokers but it was not confirmed at the protein level. A significantly greater thickness of the cellular epidermis and SC was observed in the skin of smokers (Fig. 2).

### Composition and organization of skin barrier lipids

**Figure 3:**
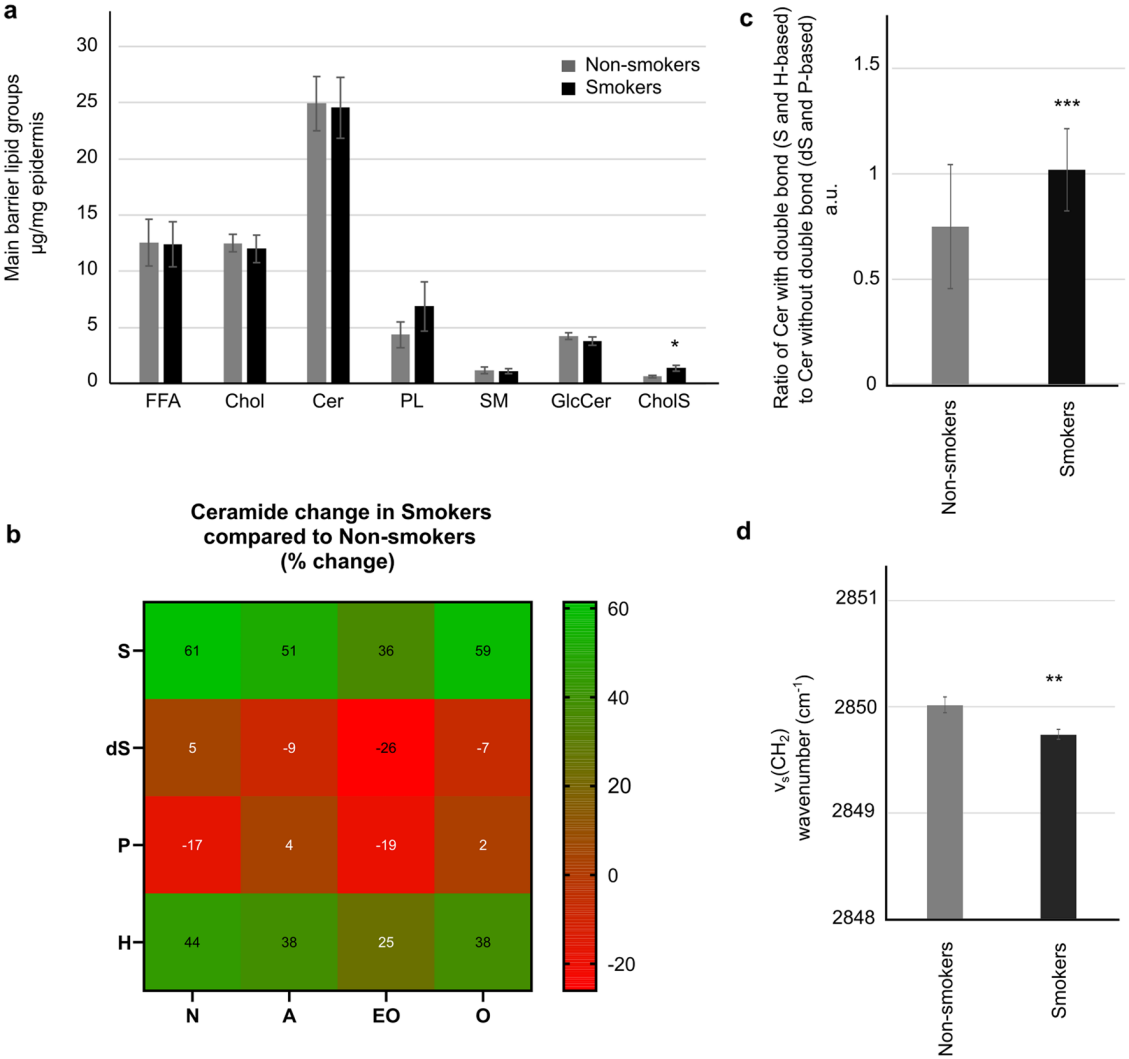
The skin barrier lipids composition and organization in the skin of smokers and non-smokers. (**a**) Quantification of the main skin barrier lipid groups and their precursors by HPTLC (FFA, free fatty acids; Chol, cholesterol; Cer, ceramide; PL, phospholipids; SM, sphingomyelin; GlcCer, glucosylceramides; CholS, cholesterol sulfate). (**b**) Percentage changes of ceramides subclasses in smokers compared to nonsmokers (LC–MS^2^). (**c**) Ratio of ceramides with double bonds at position 4 of the sphingoid base to ceramides without such double bond (LC–MS^2^). (**d**) Conformation of the skin barrier lipids determined by infrared spectroscopy.

SC lipids in smokers and non-smokers were quantified using HPTLC based on authentic external standards, which allows simple quantification of the SC lipid classes ceramides, free fatty acids, cholesterol, and cholesterol sulfate. Comparable contents of the major SC barrier lipid classes were found in both groups of volunteers (Fig. 3a), except for cholesterol sulfate, as a direct precursor of cholesterol, which was significantly increased in smokers by 117% (Fig. 3a). The precursors of ceramides (sphingomyelin, glucosylceramides) were also unaffected by long-term smoking although an increasing trend in the content of phospholipids (average increase of 58%) in smokers compared to non-smokers was observed.

To gain a deeper insight into the ceramide class, which is the most abundant and essential lipid class in SC, LC–MS^2^ was used to precisely quantify individual ceramide subclasses including their chain length distribution. The ceramides are classified based on the structure of their polar headgroup (sphingosine S, dihydrosphingosine dS, phytosphingosine P and 6-hydroxysphingosine H) and a substitution of their acyl chain (non-substituted N, α-hydroxylated A, ω-esterified EO or ω-hydroxylated O) (Supplementary Fig. S1;^16^. For the purposes of this project, all quantified ceramides had an 18-carbon sphingoid base and a chain length of fatty acyls between 16 and 28 carbons (very long chain) and between 28 and 36 carbons (ultralong chain). Average acyl chain lengths within individual ceramide subclasses were rather comparable between the two groups with a trend to a decreased average chain length in smokers. However, this observed decrease, although significant in some ceramides, was rather subtle. The final average chain lengths reached values between 24 and 26 carbons for very long chains and 30–32 carbons for the ultralong chains (particular chain lengths are shown in Supplementary Table S4), which is in accordance with the literature^17^. The distribution of chain lengths within individual ceramide subclasses was very similar for all very long chain ceramides (example shown in Supplementary Fig. S2).

LC–MS^2^ quantification revealed that individual ceramide subclasses were not equally represented in smokers and nonsmokers (percentage change is shown in Fig. 3b and Supplementary Table S5 lists the measured quantities). Certain ceramide subclasses, especially those derived from sphingosine and 6-hydroxysphingosine (both containing a double bond in their polar headgroup) were highly elevated in smokers compared to nonsmokers. On the other hand, ceramide subclasses derived from dihydrosphingosine and phytosphingosine were unchanged or decreased. When quantified together, the ratio of ceramides with double bond (S and H-based) to ceramides without double bond (dS and P-based) increased by approx. 36% and this difference was statistically significant (Fig. 3c).

To determine the effect of long-term cigarette smoking on the acyl chain conformation and lateral arrangement of the skin barrier lipids infrared spectra were measured. The value of methylene symmetric vibration below 2850 cm^–1^ indicates ordered lipid chains, while increasing wavenumbers signal chain disorder^18^. Our results showed rather ordered lipid chains in the two groups of volunteers (Fig. 3d), with a shift to lower values in the skin of smokers suggesting more compact packing of the predominant all trans chains.

## Discussion

In our study, we found significantly elevated expression of the skin barrier proteins filaggrin and loricrin in smokers and a trend of increased values for involucrin. In accordance with these results, upregulation of filaggrin was also observed in oral keratinocytes of smokers^19^, in keratinocyte cell cultures^19, 20^ or in reconstructed human epidermis^21^ exposed to cigarette smoke extract, and porcine skin explants exposed to cigarette smoke (our unpublished data). On the other hand, opposite results, downregulation of filaggrin was observed in reconstructed human epidermis exposed to cigarette smoke as well^22^. Concerning KLK7 expression, which was found unchanged in the skin of smokers in our study, Rajagopalan et al.^20^ observed its upregulation in the cell cultures treated with cigarette smoke extract. TG1 has not been studied in relation to cigarette smoke yet.

Stimulation of keratinocyte differentiation observed in our study was accompanied by the increased thickness of the cellular epidermis and SC. In accordance with our results, Yazdanparast has shown that the thickness of the epidermis was slightly higher in smokers although without statistical significance^23^. On the other hand, Sandby-Moller showed that SC thickness negatively correlated with the number of years of smoking and no relationship with current smoking status was found^24^.

Filaggrin, loricrin and involucrin are members of the epidermal differentiation complex, a cluster of genes involved in the terminal differentiation of keratinocytes. They are commonly upregulated as an adaptive response of the skin to harmful factors including environmental toxins such as PAHs present in large quantities in cigarette smoke^25^. Therefore, PAHs may contribute, at least partly, to the observed hyperkeratinization in the skin of smokers.

The action of PAHs is mediated by the aryl hydrocarbon receptor (AhR)^25^. AhR is a ligand-dependent transcription factor that participates in a whole range of biological processes including the metabolism of xenobiotics, cell differentiation, inflammation, melanogenesis and carcinogenesis^26^. The temporary activation of AhR by PAHs may be beneficial for the skin barrier reinforcement whereas sustained AhR activation is rather associated with deleterious effects^27^. The complex role of AhR in the skin is currently heavily debated^27^.

Another possible contributor to the observed cigarette smoking-induced epidermal hyperkeratinization is nicotine which has also been shown to increase various differentiation markers including filaggrin, involucrin, TG1 in keratinocytes^19^ mediated by the activation of nicotinic acetylcholine receptors abundant in the skin^28^. Subcutaneous injection of high doses of nicotine in mice achieving plasma concentrations similar to those of heavy smokers also increased epidermal thickness although lower nicotine doses simulating the plasma levels of lighter smokers had the opposite effect^29^.

In the case of the effect of cigarette smoking on the skin barrier lipids, the studies usually evaluated the direct effect of cigarette smoke on the skin where significant lipid peroxidation was accompanied by skin barrier disruption and elevated TEWL^6^. However, to the best of our knowledge, no lipidomic analysis has been performed in the skin of long-term smokers, especially in places not directly exposed to smoke, to compare our results.

The HPTLC quantification of epidermal lipids revealed significantly increased cholesterol sulfate in the skin of smokers. Although cholesterol sulfate is present in the epidermis in minor amounts, it plays an important role in epidermal differentiation, skin barrier function and desquamation^30^. The sulfation of cholesterol occurs in association with later phases of keratinocyte differentiation. For this reason, cholesterol sulfate commonly serves as a differentiation marker, but it also acts as a signaling molecule capable of induction of this process itself^30^. The level of cholesterol sulfate was shown to correlate with some keratinization proteins such as filaggrin^31^. Therefore, the elevated levels of cholesterol sulfate found in the skin of smokers in our study may be associated with the observed upregulation of epidermal differentiation. Dysregulation of cholesterol sulfate desulfation has been implicated in various skin diseases such as X-linked ichthyosis where high levels of cholesterol sulfate are thought to contribute to the impairment of the skin barrier function and desquamation^30^. Higher levels of cholesterol sulfate might, therefore, have a negative effect on the skin barrier function of smokers. Based on the quantification results, it is not possible to distinguish whether the increased content of cholesterol sulfate is caused by enhanced biosynthesis or insufficient degradation.

It is well-documented that ceramide acyl chain length significantly affects the skin barrier function. Several studies reported that short-chain ceramides were associated with increased permeability as shown in model skin lipid membranes in vitro^32^ or in patients with atopic dermatitis^33^. In the latter study, reduction in the ceramide acyl chain length had a much stronger impact on the skin barrier function than did the changes in ceramide subclass levels; and TEWL increased proportionally with decreasing chain length. It was also found that UV exposure induces a reduction in ceramide acyl chain length which might contribute to UV-induced skin barrier disruption^34^. Whether the significant, but rather subtle reduction in the acyl chain length observed in the skin of smokers in this study could affect the overall barrier function remains unclear.

Targeted LC–MS^2^ analysis revealed that ceramides based on sphingosine and 6-hydroxysphingosine were highly elevated in smokers. Both these ceramides contain a double bond in their polar head structure. Concerning the biological impact of the presence of C4 double bonds in the sphingoid base of ceramides, the increased number of the unsaturated ceramides was found in psoriasis^16^ and atopic dermatitis patients^35^. Both these diseases have impaired barrier function, suggesting a link to these unsaturated ceramides. However, Skolova et al.^32^ found no differences in the permeability, lipid chain order and packing of multilamellar skin lipid membranes containing either ceramides or corresponding dihydroceramides with C24 acyl chain length. Other studies then showed that unsaturated ceramides promoted slightly tighter packing associated with lower SC permeability in comparison to the corresponding dihydroceramides^36^. During ceramide biosynthesis the double bond in sphingosine is introduced by a desaturase DEGS1^37^. The final step in the 6-hydroxysphingosine biosynthesis has not been elucidated yet, but it is expected that a similar enzyme is involved in this process. Based on the obtained results, we can speculate that cigarette smoking may enhance the function of these desaturases, but the role of the presence of the C4 double bond in the sphingoid backbone of ceramide in the skin barrier function needs to be further elucidated.

Oxidative damage of the skin lipids directly exposed to cigarette smoke has been shown to disorder lipid acyl chains associated with skin barrier disruption^6^. However, in the volar forearms of smokers where the oxidative stress is less prominent than in more exposed areas such as the face, our results showed that the lipids were highly ordered and there was even a shift to lower methylene frequencies in comparison to the non-smokers suggesting more compact packing. A higher abundance of C4 unsaturated ceramides may have contributed to this effect as discussed above although the results of the studies are still inconclusive in this regard (Školová et al.^39^). On the other hand, the elevated levels of ceramides with shorter acyl chains, previously associated with less tight packing^38^ did not have a significant negative impact on the lipid organization in the skin of smokers. Tighter skin barrier lipid packing has also been shown to be due to lower SC hydration^39^ which has been found in the skin of smokers in several studies mainly in areas with low sebum levels including the volar forearm^4, 7, 8, 23^. Therefore, lower skin hydration could be another factor contributing to the tighter skin barrier lipid packing in the skin of smokers observed in this study.

Taken together, the results of this study showed no dramatic detrimental effects of long-term cigarette smoking on the key skin barrier components suggesting that the overall barrier function in the volar forearm skin is probably not significantly compromised or the negative effects may have been compensated by the stimulation of the epidermal differentiation in the studied group of smokers. This assumption is consistent with TEWL determined in the volar forearm skin of smokers, which was similar to nonsmokers ^7, 8^. The sun-protected, volar forearm in this case represents the area affected primarily by the systemic effect of cigarette smoke with minimal direct influence of smoke or chronic UV radiation exposure. On the other hand, quite different results have been observed in the skin directly exposed to the smoke such as the face. A large body of evidence showed that direct skin exposure to cigarette smoke containing a large number of oxidants and radical species^40^ leads to lipid peroxidation^41^ associated with the skin barrier impairment demonstrated by the increased TEWL in various experimental models^5, 6^ as well as in the cheek of smokers^4^. And although the stimulation of epidermal differentiation as a compensatory process has also been shown upon direct skin exposure to the smoke as discussed above, it was probably not sufficient to compensate for the high levels of oxidative damage.

## Conclusion

In this study, we investigated differences in the skin barrier proteins and lipids between long-term smokers and non-smokers. In smoker´s volar forearm skin, we found increased epidermal differentiation, which may represent a compensatory mechanism induced as an adaptive response to environmental toxins such as PAHs present in cigarette smoke, or nicotine, both of which have the ability to stimulate keratinocyte differentiation.

Concerning skin barrier lipids, comparable contents of the major SC barrier lipid classes and also precursors of ceramides were found in both groups of volunteers, except for cholesterol sulfate and phospholipids, which were increased in smokers. Also, average acyl chain lengths within individual ceramide subclasses were rather comparable between the two groups with a trend to a decreased average chain length in smokers. However, this observed decrease, although significant in some ceramides, was rather subtle. Quantification revealed that the ratio of ceramides with double bond (S and H-based) to ceramides without double bond (dS and P-based) increased by approx. 36% in smokers compared to nonsmokers and this difference was statistically significant. Ordered lipid chains in the two groups of volunteers was found, with a shift to lower values of methylene symmetric vibration in the skin of smokers suggesting more compact packing of the predominant all trans chains.

It seems that the overall effect of cigarette smoking on the skin barrier function depends on the balance between oxidative damage and the counteracting compensatory processes. In the face, for instance, direct exposure of the skin to smoke induces high levels of oxidative stress associated with the skin barrier disruption, whereas in the less exposed areas such as the volar forearm, the compensatory processes may maintain the barrier function. Nevertheless, the capacity of the compensatory processes may be insufficient in smokers with skin diseases or in the elderly, which warrants further studies for a better understanding of the mechanisms underlying the effects of long-term cigarette smoking on the skin barrier function and its contribution to various skin diseases.

## Supplementary Information

Supplementary Information.

## Data Availability

“The datasets generated and/or analysed during the current study are available in the [FIGSHARE] repository, [10.6084/m9.figshare.2269316]”.

## Abbreviations

AhR: Aryl hydrocarbon receptor
HPTLC: High-performance thin-layer chromatography
KLKs: Kallikrein-related peptidases
PAHs: Polycyclic aromatic hydrocarbons
qRT-PCR: Quantitative, real-time reverse transcription PCR
SC: Stratum corneum
TEWL: Transepidermal water loss
TG: Transglutaminase

## References

[1] Prieux R, Eeman M, Rothen-Rutishauser B, Valacchi G (2020). Mimicking cigarette smoke exposure to assess cutaneous toxicity. Toxicol. In Vitro.

[2] Freiman A, Bird G, Metelitsa AI, Barankin B, Lauzon GJ (2004). Cutaneous effects of smoking. J. Cutan. Med. Surg..

[3] Antonov D, Schliemann S, Elsner P (2016). Methods for the assessment of barrier function. Curr Probl Dermatol.

[4] Muizzuddin N, Marenus K, Vallon P, Maes D (1997). Effect of cigarette smoke on skin. J. Soc. Cosmet. Chem..

[5] Pavlou P, Rallis M, Deliconstantinos G, Papaioannou G, Grando S (2009). In-vivo data on the influence of tobacco smoke and UV light on murine skin. Toxicol. Ind. Health.

[6] Percoco G (2021). Impact of cigarette smoke on physical-chemical and molecular proprieties of human skin in an ex vivo model. Exp. Dermatol..

[7] Xin S (2016). Heavy cigarette smokers in a Chinese population display a compromised permeability barrier. Biomed. Res. Int..

[8] Majewski S (2017). Skin condition and its relationship to systemic inflammation in chronic obstructive pulmonary disease. Int. J. Chronic Obstr. Pulm. Dis..

[9] Kantor R, Kim A, Thyssen JP, Silverberg JI (2016). Association of atopic dermatitis with smoking: A systematic review and meta-analysis. J. Am. Acad. Dermatol..

[10] Richer V (2016). Psoriasis and smoking: A systematic literature review and meta-analysis with qualitative analysis of effect of smoking on psoriasis severity. J. Cutan. Med. Surg..

[11] Candi E, Schmidt R, Melino G (2005). The cornified envelope: A model of cell death in the skin. Nat. Rev. Mol. Cell Biol..

[12] Voegeli R, Rawlings AV, Lodén M, Maibach HI (2012). Desquamation: It is almost all about proteases. Treatment of Dry Skin Syndrome: The Art and Science of Moisturizers.

[13] Svoboda M (2017). Comparison of suction blistering and tape stripping for analysis of epidermal genes, proteins and lipids. Arch. Dermatol. Res..

[14] Schindelin J (2012). Fiji: an open-source platform for biological-image analysis. Nat. Methods.

[15] Pullmannová P (2019). Long and very long lamellar phases in model stratum corneum lipid membranes. J. Lipid Res..

[16] Motta S (1993). Ceramide composition of the psoriatic scale. Biochim. Biophys. Acta.

[17] Suzuki M, Ohno Y, Kihara A (2022). Whole picture of human stratum corneum ceramides, including the chain-length diversity of long-chain bases. J. Lipid Res..

[18] Boncheva M, Damien F, Normand V (2008). Molecular organization of the lipid matrix in intact Stratum corneum using ATR-FTIR spectroscopy. Biochim. Biophys. Acta (BBA) Biomembr..

[19] Renò F, Rocchetti V, Migliario M, Rizzi M, Cannas M (2011). Chronic exposure to cigarette smoke increases matrix metalloproteinases and Filaggrin mRNA expression in oral keratinocytes: Role of nicotine stimulation. Oral Oncol..

[20] Rajagopalan P (2016). How does chronic cigarette smoke exposure affect human skin? A global proteomics study in primary human keratinocytes. OMICS.

[21] Hubaux, R., Weisgerber, F. & Salmon, M. In vitro assays to study the effects of air pollutants on skin: Exposure to urban dust and cigarette smoke extract. *IFSCC 2015* (2015).

[22] Lecas S (2016). In vitro model adapted to the study of skin ageing induced by air pollution. Toxicol. Lett..

[23] Yazdanparast T (2019). Cigarettes smoking and skin: A comparison study of the biophysical properties of skin in smokers and non-smokers. Tanaffos.

[24] Sandby-Møller J, Poulsen T, Wulf HC (2003). Epidermal thickness at different body sites: Relationship to age, gender, pigmentation, blood content, skin type and smoking habits. Acta Derm. Venereol..

[25] Furue M (2015). Gene regulation of filaggrin and other skin barrier proteins via aryl hydrocarbon receptor. J. Dermatol. Sci..

[26] Esser C, Bargen I, Weighardt H, Haarmann-Stemmann T, Krutmann J (2013). Functions of the aryl hydrocarbon receptor in the skin. Semin. Immunopathol..

[27] van den Bogaard EH, Perdew GH (2021). The enigma of aryl hydrocarbon receptor activation in skin: Interplay between ligands, metabolism and bioavailability. J. Invest. Dermatol..

[28] Grando SA (1996). Activation of keratinocyte nicotinic cholinergic receptors stimulates calcium influx and enhances cell differentiation. J. Investig. Dermatol..

[29] Eltony SA, Ali SS (2017). Histological study on the effect of nicotine on adult male guinea pig thin skin. Anat. Cell Biol..

[30] Elias PM, Williams ML, Choi E-H, Feingold KR (2014). Role of cholesterol sulfate in epidermal structure and function: Lessons from X-linked ichthyosis. Biochim. Biophys. Acta.

[31] Hanyu O (2012). Cholesterol sulfate induces expression of the skin barrier protein filaggrin in normal human epidermal keratinocytes through induction of RORα. Biochem. Biophys. Res. Commun..

[32] Skolová B (2014). The role of the trans double bond in skin barrier sphingolipids: permeability and infrared spectroscopic study of model ceramide and dihydroceramide membranes. Langmuir.

[33] van Smeden J (2014). The importance of free fatty acid chain length for the skin barrier function in atopic eczema patients. Exp. Dermatol..

[34] Barresi R (2021). ARTICLE: Alteration to the skin barrier integrity following broad-spectrum UV exposure in an ex vivo tissue model. J. Drugs Dermatol..

[35] Janssens M (2011). Lamellar lipid organization and ceramide composition in the stratum corneum of patients with atopic eczema. J. Invest. Dermatol..

[36] Brockman H (2004). The 4,5-double bond of ceramide regulates its dipole potential, elastic properties, and packing behavior. Biophys. J ..

[37] Ternes P, Franke S, Zähringer U, Sperling P, Heinz E (2002). Identification and characterization of a sphingolipid delta 4-desaturase family. J. Biol. Chem..

[38] Školová B (2014). The role of the trans double bond in skin barrier sphingolipids: Permeability and infrared spectroscopic study of model ceramide and dihydroceramide membranes. Langmuir.

[39] Vyumvuhore R (2013). Effects of atmospheric relative humidity on stratum corneum structure at the molecular level: Ex vivo Raman spectroscopy analysis. Analyst.

[40] Valacchi G (2012). Cutaneous responses to environmental stressors. Ann. N. Y. Acad. Sci..

[41] Hergesell K, Valentová K, Velebný V, Vávrová K, Dolečková I (2022). Common cosmetic compounds can reduce air pollution-induced oxidative stress and pro-inflammatory response in the skin. Skin Pharmacol. Physiol..

