# Supplementary material for: The effect of long-term cigarette smoking on selected skin barrier proteins and lipids
Source: Sci Rep. 2023 Jul 18;13:11572. doi: 10.1038/s41598-023-38178-7 (PMC10354193; doi:10.1038/s41598-023-38178-7)
Supplement: Supplementary file 1 — Supplementary Information. [file 41598_2023_38178_MOESM1_ESM.docx]

# Supplementary

## Materials and Methods

**Quantitative, real-time RT-PCR (qRT-PCR)**

The epidermis collected by suction blister was homogenized (TissueLyser II, Qiagen, Germany) and the total RNA was isolated by the acid guanidine thiocyanate-phenol extraction method using RNAzol Reagent (Molecular Research Center, USA) according to the instructions provided by the supplier. The reverse transcription reaction was then performed with High Capacity RNA to cDNA Kit (Invitrogen, USA) according to the manufacturer’s instructions. Subsequent qPCR was performed with specific TaqMan gene expression assays (*FLG*: Hs00856927_g1; *LOR*: Hs01894962_s1; *IVL*: Hs00846307_s1; *KLK7*: Hs00192503_m1; *TGM1*: Hs00165929_m1; *RPL13A*: Hs04194366_g1; all ThermoFisher Scientific, USA) and TaqMan Fast Advanced Mastermix (Applied Biosystems, USA) according to the supplier’s recommendations in a StepOne real time PCR cycler (Applied Biosystems, USA). *RPL13A* was used as a reference gene. The data were analyzed using the 2^-ΔΔCt^ method.

**Immunofluorescence staining**

The epidermis samples collected by suction blister were fixed in 4 % paraformaldehyde overnight at 4 °C, embedded in paraffin and cross-sectioned (5 µm). The cross-sections were then deparaffinized using xylene and washed in Tris-buffered saline (TBS). Antigen retrieval was carried out with Tris/HCl EDTA buffer (10 mM Tris Base, 1 mM EDTA, 0.05 % Tween 20, pH 9) and permeability of the cell wall was increased with 0.1 % Triton-X in TBS. The cross-sections were then blocked with 10 % fetal bovine serum (FBS) in TBS-T for 60 min at RT and probed with the appropriate primary rabbit polyclonal antibodies (anti-loricrin: 1:100, ab85679; anti-involucrin: 1:100, ab53112, both Abcam, UK; anti-filaggrin: 1:1000, PA5-83128; anti-KLK7: 1:50, PA5-49707; TG1: 1:500 PA5-59088; all three Invitrogen, USA) in 10 % FBS in TBS-T overnight at 4 °C. After washing in TBS, the cross-sections were incubated with the anti-rabbit secondary antibody conjugated with Alexa Fluor 555 (1:500, A-21235, Invitrogen, USA) in 10 % FBS in TBS-T for 60 min at RT. After subsequent washing in TBS, the cross-sections were mounted in Prolong Diamond with DAPI (ThermoFisher Scientific, USA) and imaged using a Leica TCS SP8 X confocal microscope (Leica Microsystems, Germany). The images were acquired in z-stacks covering the thickness of the whole section applying 1.8 µm steps in the quantitative mode. Then, the fluorescence quantification was performed using FiJi software (Schindelin et al. 2012). The maximum projection images were segmented using fixed threshold value and mean fluorescence intensity in the segmented area was calculated. The cutoff value for the threshold was determined using a negative control image (secondary antibody only).

**High-performance thin-layer chromatography (HPTLC) of lipids**

After FTIR analysis, the extraction of the suction blister epidermal lipids was performed according Bligh and Dyer with slight modifications (Bligh and Dyer 1959). The lipids were extracted by 1 mL CHCl_3_/MeOH 2:1 (v/v) per mg of the sample for 90 min twice, extracts were combined, filtered and the solvent was evaporated under a stream of nitrogen. Then, the lipids were dried under reduced pressure overnight and stored at -20 °C. Before analysis, the extracted lipids were dissolved in 200 µL CHCl_3_/MeOH 2:1 (v/v) per mg of epidermis and 20 µL of each sample was sprayed on the silica gel 60 HPTLC plate (20 x 10 cm, Merck, Germany) using a Linomat V (Camag, Switzerland) as described previously (Vávrová et al. 2014). Standard lipids for generation of the calibration curves were purchased from Avanti Polar Lipids (USA) or synthesized by published procedures (Opálka et al. 2015; Kováčik et al. 2016) and subsequently mixed in a ratio corresponding to the composition of human SC (see Tab. S1) and analyzed along with the samples on the same HPTLC plate. The plates were developed in the automatic developing chamber ADC2 (Camag, Switzerland) with controlled humidity (33 – 36 % RH) and temperature (25 – 27°C). The major skin barrier lipids as ceramides, Chol and FFA were separated using CHCl_3_/MeOH/acetic acid 190:9:1.5 (v/v/v) mobile phase to 85 mm. To separate polar lipid precursors as SM, GlcCer, PL, and CholS, CHCl_3_/MeOH/acetic acid/H_2_O 66:25:6:3 (v/v/v/v) mobile phase was used, and the plate was developed once to 85 mm. Finally, the lipids were visualized by dipping in a derivatization reagent (7.5 % CuSO_4_, 8 % H_3_PO_4_, 10 % MeOH in water) for 10 sec and heating at 160 °C for 30 min. Quantification was performed by densitometry using a TLC scanner 3 and VisionCats software (Camag, Switzerland).

**Liquid chromatography coupled with tandem mass spectrometry (LC-MS^2^) analysis of ceramides**

Sample preparation for LCMS analysis

Protein content in the TS samples was determined by D-Squame-Scan (Clinical & Derm, USA) according to the recommendations from the manufacturer. Always 4 discs from 1 skin spot were transferred into a single 15 mL centrifuge glass tube and 100 µL of the internal standard mixture was added. The internal standard mixture contained ceramides NS (d18:1/14:0), NS (d18:1/19:0), NS (d18:1/25:0), NS (d18:1/31:0), NdS (d18:0/14:0), NP (t18:0/14:0), EOS (d18:1/h29:0/18:2) and sphingoid bases sphingosine (d14:1) and dihydrosphingosine (d17:0) in concentration of 200 nmol/L Each tube was filled with 8 mL of solvent (CHCl_3_/MeOH/H_2_O, 30:60:8 (v/v/v)) and lipids were extracted overnight at RT shaking (Pilz et al. 2022). Subsequently, the samples were centrifuged (10 min, 3500 RPM) and the supernatant (7 mL) was collected into a separate tube. The test tubes with discs were refilled with 7 mL of additional solvent (CHCl_3_/MeOH/H_2_O 30:60:8 (v/v/v)) and placed in an ultrasonic bath (45 °C) for 3 min and then left for additional 12 min in the bath. After centrifugation (10 min, 3500 RPM), 7 mL of the supernatant was collected and combined with the previously taken supernatants. The lipid extracts were dried under a gentle stream of nitrogen at 43 °C. The dry lipid extract was reconstituted using 1.5 mL of CHCl_3_/MeOH 1:9 (v/v), sonicated for 3 minutes, filtered through 0.20 µm PTFE filters and transferred into 1.5 mL vials for LCMS analysis.

LC-MS^2^ analysis of SC ceramides

Ceramides and sphingoid bases were analyzed using Shimadzu prominence HPLC instrument and Shimadzu LCMS 8050 instrument (both Shimadzu, Japan). Ceramides were separated on 15 cm Ascentis C18 column (15 cm × 2.1 mm; 3 µm, Supelco, USA) using a gradient between 57 % solvent A (50 % MeOH, 50 % water) and 99 % solvent B (99 % isopropanol, 1 % MeOH), both containing 10 mM ammonium formate and 0.1 % formic acid as additives (see Tab. S2) at a flow rate of 0.2 mL/min at 30 °C. Lipids analyzed via LCMS with their corresponding transitions and retention times are listed in Tab. S3 and were detected using multi-reaction monitoring (MRM). The quantification was performed based on the previously-mentioned internal standards and the following external standards: Ceramide NS(d18:1;24:0), NdS(d18:0;24:0), NP(t18:0;24:0), NH(t18:1;24:0), EOS(d18:1;h32:0;18:2), EOP(t18:1;h32:0;18:2), EOdS(d18:0;h32:0;18:2), OS(d18:1;h32:0), OdS(d18:0;h32:0), OP(t18:0;h32:0), AS(d18:1;h24:0), AdS(d18:0;h24:0), AP(t18:0;h24:0) and sphingoid bases sphingosine (d18:1), dihydrosphingosine (d18:0) and phytosphingosine (t18:0). Sphingoid bases were purchased from Avanti Polar Lipids (Alabaster, USA), ceramides were prepared by a direct acylation of sphingoid bases with appropriate fatty acids using *N*-(3-dimethylaminopropyl)-*N′*-ethylcarbodiimide and 1-hydroxybenzotriazole hydrate. ω‑esterificated and ω-hydroxylated ceramides were prepared according to a modified procedure based on Opálka et al. (Opálka et al. 2015). For quantification, the correction for increasing sphingolipid chain length was taken into account.

| Lipid standard | Calibration curve range [µg] |
| --- | --- |
| Cholesterol | 0.50 – 10.00 |
| Lignoceric acid | 0.40 – 8.00 |
| Ceramide (Cer) EOS | 0.03 – 0.60 |
| Cer NS | 0.20 – 4.00 |
| Cer EOP | 0.04 – 0.80 |
| Cer NP | 0.10 – 2.00 |
| Cer AS | 0.08 – 1.60 |
| Cer AP | 0.08 – 1.60 |
| Cholesterol sulfate | 0.10 – 2.50 |
| GlucosylCer | 0.20 –5.00 |
| Sphingomyelin | 0.20 –5.00 |
| L-α-phosphatidylcholine | 0.50 –12.50 |

*Supplementary Tab. S1. Concentration of standards for HPTLC quantification.*

| Time (min) | Pump B concentration (%) |
| --- | --- |
| 0.02 | 43 |
| 0.04 | 50 |
| 4 | 70 |
| 23 | 99 |
| 28 | 99 |
| 29 | 43 |
| 32 | 43 |

*Supplementary Tab. S2. HPLC method for LC-MS^2^ quantification of ceramides.*


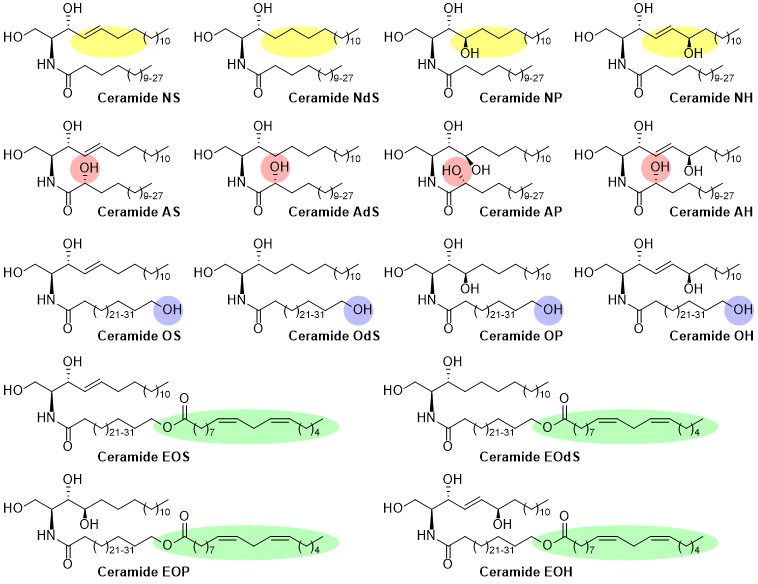


*Supplementary Fig.S1. Chemical structures of the quantified ceramides.*

| Cer | MRM transition | RT | Cer | MRM transition | RT |
| --- | --- | --- | --- | --- | --- |
| NS (18_1,14_0) | 510.6000>264.2700 | 11.51 | EOS (18_1,26_0,18_2) | 956.9100>264.2700 | 22.3 |
| NS (18_1,16_0) | 538.6300>264.2700 | 13.009 | EOS (18_1,28_0,18_2) | 984.9400>264.2700 | 22.937 |
| NS (18_1,18_0) | 566.6600>264.2700 | 14.388 | EOS (18_1,29_0,18_2) | 998.9500>264.2700 | 23.191 |
| NS (18_1,19_0) | 580.6700>264.2700 | 15.499 | EOS (18_1,30_0,18_2) | 1012.9600>264.2700 | 23.568 |
| NS (18_1,20_0) | 594.6800>264.2700 | 16.111 | EOS (18_1,32_0,18_2) | 1040.9900>264.2700 | 24.346 |
| NS (18_1,22_0) | 622.7100>264.2700 | 17.605 | EOS (18_1,34_0,18_2) | 1069.0200>264.2700 | 24.972 |
| NS (18_1,24_0) | 650.7400>264.2700 | 18.886 | EOS (18_1,36_0,18_2) | 1097.0500>264.2700 | 25.6 |
| NS (18_1,26_0) | 678.7700>264.2700 | 20.084 | EOdS (18_0,26_0,18_2) | 958.9200>284.5000 | 22.1 |
| NS (18_1,28_0) | 706.8000>264.2700 | 21.173 | EOdS (18_0,28_0,18_2) | 986.9500>284.5000 | 22.953 |
| NS (18_1,30_0) | 734.8300>264.2700 | 22.169 | EOdS (18_0,30_0,18_2) | 1014.9800>284.5000 | 23.881 |
| NS (18_1,31_0) | 748.8400>264.2700 | 22.633 | EOdS (18_0,32_0,18_2) | 1043.0100>284.5000 | 24.687 |
| NS (18_1,32_0) | 762.8500>264.2700 | 22.911 | EOdS (18_0,34_0,18_2) | 1071.0400>284.5000 | 25.45 |
| NdS (18_0,14_0) | 512.5000>284.2900 | 11.887 | EOdS (18_0,36_0,18_2) | 1099.0700>284.5000 | 26.3 |
| NdS (18_0,16_0) | 540.5300>284.2900 | 13.286 | EOP (18_0,26_0,18_2) | 974.9100>282.4000 | 21.55 |
| NdS (18_0,18_0) | 568.5600>284.2900 | 14.924 | EOP (18_0,28_0,18_2) | 1002.9400>282.4000 | 22.315 |
| NdS (18_0,20_0) | 596.5900>284.2900 | 16.435 | EOP (18_0,30_0,18_2) | 1030.9700>282.4000 | 23.091 |
| NdS (18_0,22_0) | 624.6200>284.2900 | 17.914 | EOP (18_0,32_0,18_2) | 1059.0000>282.4000 | 23.883 |
| NdS (18_0,24_0) | 652.6500>284.2900 | 19.127 | EOP (18_0,34_0,18_2) | 1087.0300>282.4000 | 24.692 |
| NdS (18_0,26_0) | 680.6800>284.2900 | 20.223 | EOP (18_0,36_0,18_2) | 1115.0600>282.4000 | 25.5 |
| NdS (18_0,28_0) | 708.7100>284.2900 | 21.259 | EOH isd (18_1,26_0,18_2) | 954.8900>280.3000 | 20.459 |
| NdS (18_0,30_0) | 736.7400>284.2900 | 22.259 | EOH isd (18_1,28_0,18_2) | 982.9200>280.3000 | 21.369 |
| NdS (18_0,32_0) | 764.7700>284.2900 | 23.331 | EOH isd (18_1,30_0,18_2) | 1010.9500>280.3000 | 22.225 |
| NP (18_0,14_0) | 528.4900>282.2800 | 10.575 | EOH isd (18_1,32_0,18_2) | 1038.9800>280.3000 | 23.091 |
| NP (18_0,16_0) | 556.5200>282.2800 | 11.994 | EOH isd (18_1,34_0,18_2) | 1067.0100>280.3000 | 23.931 |
| NP (18_0,18_0) | 584.5500>282.2800 | 13.556 | EOH isd (18_1,36_0,18_2) | 1095.0400>280.3000 | 25.074 |
| NP (18_0,20_0) | 612.5800>282.2800 | 15.111 | OS (18_1,26_0) | 694.6700>264.3000 | 12.85 |
| NP (18_0,22_0) | 640.6100>282.2800 | 16.742 | OS (18_1,28_0) | 722.7000>264.3000 | 14.287 |
| NP (18_0,24_0) | 668.6400>282.2800 | 17.956 | OS (18_1,30_0) | 750.7300>264.3000 | 15.625 |
| NP (18_0,26_0) | 696.6700>282.2800 | 19.259 | OS (18_1,32_0) | 778.7600>264.3000 | 17.038 |
| NP (18_0,28_0) | 724.7000>282.2800 | 20.386 | OS (18_1,34_0) | 806.7900>264.3000 | 18.147 |
| NP (18_0,30_0) | 752.7300>282.2800 | 21.52 | OS (18_1,36_0) | 834.8200>264.3000 | 19.25 |
| NP (18_0,32_0) | 780.7600>282.2800 | 22.479 | OdS (18_0,26_0) | 696.6900>284.5000 | 14.25 |
| NH isd (18_1,14_0) | 508.4700>280.3000 | 9.161 | OdS (18_0,28_0) | 724.7200>284.5000 | 15.3 |
| NH isd (18_1,16_0) | 536.5000>280.3000 | 10.498 | OdS (18_0,30_0) | 752.7500>284.5000 | 16.324 |
| NH isd (18_1,18_0) | 564.5300>280.3000 | 11.999 | OdS (18_0,32_0) | 780.7800>284.5000 | 17.371 |
| NH isd (18_1,20_0) | 592.5600>280.3000 | 13.611 | OdS (18_0,34_0) | 808.8100>284.5000 | 18.4 |
| NH isd (18_1,22_0) | 620.5900>280.3000 | 14.993 | OdS (18_0,36_0) | 836.8400>284.5000 | 19.45 |
| NH isd (18_1,24_0) | 648.6200>280.3000 | 16.429 | OP (18_0,26_0) | 712.6800>282.4000 | 12.4 |
| NH isd (18_1,26_0) | 676.6500>280.3000 | 17.748 | OP (18_0,28_0) | 740.7100>282.4000 | 13.588 |
| NH isd (18_1,28_0) | 704.6800>280.3000 | 18.983 | OP (18_0,30_0) | 768.7400>282.4000 | 14.802 |
| NH isd (18_1,30_0) | 732.7100>280.3000 | 20.142 | OP (18_0,32_0) | 796.7700>282.4000 | 16.171 |
| NH isd (18_1,32_0) | 760.7400>280.3000 | 21.136 | OP (18_0,34_0) | 824.8000>282.4000 | 17.55 |
| AS isd (18_1,14_0) | 508.4700>264.5000 | 10.733 | OP (18_0,36_0) | 852.8300>282.4000 | 18.9 |
| AS isd (18_1,16_0) | 536.5000>264.5000 | 12.101 | OH isd (18_1,26_0) | 692.6600>280.3000 | 11.1 |
| AS isd (18_1,18_0) | 564.5300>264.5000 | 13.664 | OH isd (18_1,28_0) | 720.6900>280.3000 | 12.36 |
| AS isd (18_1,20_0) | 592.5600>264.5000 | 15.3 | OH isd (18_1,30_0) | 748.7200>280.3000 | 13.632 |
| AS isd (18_1,22_0) | 620.5900>264.5000 | 16.608 | OH isd (18_1,32_0) | 776.7500>280.3000 | 15.04 |
| AS isd (18_1,24_0) | 648.6200>264.5000 | 17.958 | OH isd (18_1,34_0) | 804.7800>280.3000 | 16.4 |
| AS isd (18_1,26_0) | 676.6500>264.5000 | 19.181 | OH isd (18_1,36_0) | 832.8100>280.3000 | 17.8 |
| AS isd (18_1,28_0) | 704.6800>264.5000 | 20.307 | S (14_1) | 244.2300>196.2100 | 2.723 |
| AS isd (18_1,30_0) | 732.7100>264.5000 | 21.517 | S (16_1) | 272.2600>224.2400 | 3.482 |
| AS isd (18_1,32_0) | 760.7400>264.5000 | 22.436 | S (18_1) | 300.2900>252.2700 | 4.586 |
| AdS (18_0,14_0) | 528.5000>284.5000 | 11.303 | S (20_1) | 328.3200>280.3000 | 5.662 |
| AdS (18_0,16_0) | 556.5300>284.5000 | 12.711 | S (22_1) | 356.3500>308.3300 | 6.844 |
| AdS (18_0,18_0) | 584.5600>284.5000 | 14.27 | S (24_1) | 384.3800>336.3600 | 7.846 |
| AdS (18_0,20_0) | 612.5600>284.5000 | 15.8 | S (26_1) | 412.4100>364.3900 | 8.939 |
| AdS (18_0,22_0) | 640.6200>284.5000 | 17.24 | S (28_1) | 440.4400>392.4200 | 10.078 |
| AdS (18_0,24_0) | 668.6500>284.5000 | 18.539 | S (30_1) | 468.4700>420.4500 | 11.2 |
| AdS (18_0,26_0) | 696.6800>284.5000 | 19.703 | S (32_1) | 496.5000>448.4800 | 12.494 |
| AdS (18_0,28_0) | 724.7100>284.5000 | 20.82 | dS (14_0) | 246.2500>198.2300 | 2.3 |
| AdS (18_0,30_0) | 752.7400>284.5000 | 21.9 | dS (16_0) | 274.2800>226.2600 | 3.523 |
| AdS (18_0,32_0) | 780.7700>284.5000 | 22.95 | dS (17_0) | 288.2900>240.2700 | 4.351 |
| AP (18_0,14_0) | 544.5000>282.4000 | 10.071 | dS (18_0) | 302.3000>254.2800 | 4.867 |
| AP (18_0,16_0) | 572.5300>282.4000 | 11.382 | dS (20_0) | 330.3300>282.3100 | 5.973 |
| AP (18_0,18_0) | 600.5600>282.4000 | 12.906 | dS (22_0) | 358.3600>310.3400 | 7.122 |
| AP (18_0,20_0) | 628.5900>282.4000 | 14.399 | dS (24_0) | 386.3900>338.3700 | 8.175 |
| AP (18_0,22_0) | 656.6200>282.4000 | 15.839 | dS (26_0) | 414.4200>366.4000 | 9.326 |
| AP (18_0,24_0) | 684.6500>282.4000 | 17.269 | dS (28_0) | 442.4500>394.4300 | 10.641 |
| AP (18_0,26_0) | 712.6800>282.4000 | 18.524 | dS (30_0) | 470.4800>422.4600 | 12.134 |
| AP (18_0,28_0) | 740.7100>282.4000 | 19.717 | dS (32_0) | 498.5100>450.4900 | 13.456 |
| AP (18_0,30_0) | 768.7400>282.4000 | 21.02 | P (14_0) | 264.2400>214.2200 | 2.272 |
| AP (18_0,32_0) | 796.7700>282.4000 | 21.87 | P (16_0) | 290.2700>242.2500 | 2.934 |
| AH (18_1,14_0) | 524.4700>280.3000 | 8.3 | P (18_0) | 318.3000>270.2800 | 3.998 |
| AH (18_1,16_0) | 552.5000>280.3000 | 9.865 | P (20_0) | 346.3300>298.3100 | 5.187 |
| AH (18_1,18_0) | 580.5300>280.3000 | 11.324 | P (22_0) | 374.3600>326.3400 | 6.296 |
| AH (18_1,20_0) | 608.5600>280.3000 | 12.469 | P (24_0) | 402.3900>354.3700 | 7.88 |
| AH (18_1,22_0) | 636.5900>280.3000 | 14.153 | P (26_0) | 430.4200>382.4000 | 8.981 |
| AH (18_1,24_0) | 664.6200>280.3000 | 15.65 | P (28_0) | 458.4500>410.4300 | 0.001 |
| AH (18_1,26_0) | 692.6500>280.3000 | 17.012 | P (30_0) | 486.4800>438.4600 | 0.001 |
| AH (18_1,28_0) | 720.6800>280.3000 | 18.257 | P (32_0) | 514.5100>466.4900 | 0.001 |
| AH (18_1,30_0) | 748.7100>280.3000 | 19.4 | H (14_1) | 260.2200>212.2000 | 3.3 |
| AH (18_1,32_0) | 776.7400>280.3000 | 20.35 | H (16_1) | 288.2500>240.2300 | 4.353 |
|  |  |  | H (18_1) | 316.2800>268.2600 | 5.43 |
|  |  |  | H (20_1) | 344.3100>296.2900 | 6.531 |
|  |  |  | H (22_1) | 372.3400>324.3200 | 7.626 |
|  |  |  | H (24_1) | 400.3700>352.3500 | 8.744 |
|  |  |  | H (26_1) | 428.4000>380.3800 | 9.935 |
|  |  |  | H (28_1) | 456.4300>408.4100 | 11.35 |
|  |  |  | H (30_1) | 484.4600>436.4400 | 12.803 |
|  |  |  | H (32_1) | 512.4900>464.4700 | 14.25 |

*Supplementary Tab. S3. LC-MS^2^ MRM transitions and retention times of the quantified ceramides.*

## Results

|  | Cer average chain length [number of C] | | | |
| --- | --- | --- | --- | --- |
|  | Non-smokers | | Smokers | |
| Cer | Mean | SD | Mean | SD |
| NS | 25.20 | 0.39 | 25.01 | 0.39 |
| NdS | 24.58 | 0.39 | 24.37 | 0.33 |
| NP | 25.49 | 0.32 | 25.43 | 0.21 |
| NH | 25.94 | 0.13 | 25.90 | 0.10 |
| AS | 24.89 | 0.25 | 24.79 | 0.12 |
| AdS | 24.46 | 0.84 | 24.55 | 0.80 |
| AP | 24.50 | 0.17 | 24.31 | 0.12 |
| AH | 25.24 | 0.16 | 25.15 | 0.12 |
| EOS | 30.89 | 0.15 | 30.87 | 0.14 |
| EOdS | 30.44 | 0.49 | 30.47 | 0.54 |
| EOP | 30.96 | 0.50 | 30.77 | 0.31 |
| EOH | 31.48 | 0.34 | 31.50 | 0.24 |
| OS | 30.92 | 0.38 | 30.96 | 0.25 |
| OdS | 31.00 | 1.14 | 31.34 | 1.34 |
| OP | 31.00 | 0.87 | 31.08 | 0.75 |
| OH | 31.17 | 0.19 | 31.33 | 0.27 |

*Supplementary Tab. S4. Average chain lengths of the quantified ceramides. The results show the number of carbons in the acyl chain of ceramides.*

*Supplementary Fig. S2. Example of the percentual distribution of chain lengths within individual ceramide subclasses (Cer NdS).*

|  | Cer quantity [pmol(SL)/%A.U.(protein)] | | | |
| --- | --- | --- | --- | --- |
|  | Non-smokers | | Smokers | |
| Cer | Mean | SD | Mean | SD |
| NS | 5.04 | 3.98 | 8.13 | 6.37 |
| NdS | 6.04 | 5.92 | 6.33 | 5.01 |
| NP | 83.77 | 87.72 | 69.50 | 59.80 |
| NH | 31.67 | 24.49 | 45.65 | 29.84 |
| AS | 17.96 | 13.33 | 27.20 | 25.22 |
| AdS | 2.99 | 2.90 | 2.73 | 2.20 |
| AP | 50.30 | 46.06 | 52.29 | 50.33 |
| AH | 27.63 | 21.49 | 38.25 | 30.98 |
| EOS | 2.79 | 1.56 | 3.79 | 1.90 |
| EOdS | 0.23 | 0.21 | 0.17 | 0.11 |
| EOP | 2.88 | 2.74 | 2.33 | 1.23 |
| EOH | 3.62 | 2.43 | 4.51 | 2.41 |
| OS | 0.56 | 0.38 | 0.89 | 0.65 |
| OdS | 0.36 | 0.38 | 0.34 | 0.23 |
| OP | 1.84 | 1.12 | 1.88 | 1.31 |
| OH | 0.69 | 0.39 | 0.95 | 0.58 |

*Supplementary Tab. S5. Results of the ceramide quantification using LC-MS^2^. Values show the total concentration of individual ceramide subclasses in picomols in sample divided by the protein content in arbitrary units.*

## References

Bligh, E. G., and W. J. Dyer. 1959. ‘A Rapid Method of Total Lipid Extraction and Purification’. *Canadian Journal of Biochemistry and Physiology* 37 (8): 911–17. https://doi.org/10.1139/o59-099.

Kováčik, Andrej, Lukáš Opálka, Michaela Šilarová, Jaroslav Roh, and Kateřina Vávrová. 2016. ‘Synthesis of 6-Hydroxyceramide Using Ruthenium-Catalyzed Hydrosilylation–Protodesilylation. Unexpected Formation of a Long Periodicity Lamellar Phase in Skin Lipid Membranes’. *RSC Advances* 6 (77): 73343–50. https://doi.org/10.1039/C6RA16565F.

Opálka, Lukáš, Andrej Kováčik, Michaela Sochorová, Jaroslav Roh, Jiří Kuneš, Juraj Lenčo, and Kateřina Vávrová. 2015. ‘Scalable Synthesis of Human Ultralong Chain Ceramides’. *Organic Letters* 17 (21): 5456–59. https://doi.org/10.1021/acs.orglett.5b02816.

Pilz, Robert, Lukáš Opálka, Adam Majcher, Elisabeth Grimm, Lionel Van Maldergem, Silvia Mihalceanu, Knut Schäkel, et al. 2022. ‘Formation of Keto-Type Ceramides in Palmoplantar Keratoderma Based on Biallelic KDSR Mutations in Patients’. *Human Molecular Genetics* 31 (7): 1105–14. https://doi.org/10.1093/hmg/ddab309.

Schindelin, Johannes, Ignacio Arganda-Carreras, Erwin Frise, Verena Kaynig, Mark Longair, Tobias Pietzsch, Stephan Preibisch, et al. 2012. ‘Fiji: An Open-Source Platform for Biological-Image Analysis’. *Nature Methods* 9 (7): 676–82. https://doi.org/10.1038/nmeth.2019.

Vávrová, Kateřina, Dominika Henkes, Kay Strüver, Michaela Sochorová, Barbora Školová, Madeleine Y. Witting, Wolfgang Friess, et al. 2014. ‘Filaggrin Deficiency Leads to Impaired Lipid Profile and Altered Acidification Pathways in a 3D Skin Construct’. *Journal of Investigative Dermatology* 134 (3): 746–53. https://doi.org/10.1038/jid.2013.402.
